# Effects of Probiotics on Depressive or Anxiety Variables in Healthy Participants Under Stress Conditions or With a Depressive or Anxiety Diagnosis: A Meta-Analysis of Randomized Controlled Trials

**DOI:** 10.3389/fneur.2020.00421

**Published:** 2020-05-22

**Authors:** Limin Chao, Cui Liu, Senawin Sutthawongwadee, Yuefei Li, Weijie Lv, Wenqian Chen, Linzeng Yu, Jiahao Zhou, Ao Guo, Zengquan Li, Shining Guo

**Affiliations:** College of Veterinary Medicine, South China Agricultural University, Guangzhou, China

**Keywords:** probiotics, depression, anxiety, under stress, meta-analysis

## Abstract

**Background:** Probiotics have been associated with the treatment of depression and anxiety. However, the results reported in the literature have been inconsistent, and no meta-analysis specifically reported probiotics used on participants with varying levels of emotional state.

**Methods:** This meta-analysis aimed to study the effectiveness of probiotics on anxious or depressive symptomatology for participants under stress conditions or with a depressive or anxiety disorder diagnosis. Medline, PubMed, EMBASE, and the Cochrane Library were searched through December 2019 for randomized controlled trials (RCTs). The primary outcomes were depression and anxiety scores. Main inclusion criteria: RCTs of probiotics for participants with a mood or emotional disorder diagnosis or under stress situations; and all participants were adults (age ≥16 years); Assessed by the modified Jadad assessment scale found seven high-quality studies and three low-quality studies.

**Results:** Ten clinical trials (*n* = 685 total participants) were included based on the inclusion and exclusion criteria. All studies were assessed as low or moderate risk of bias. The meta-analysis showed that probiotics could significantly reduce the depression scale for patients with anxiety and depression, and healthy participants under stress. However, there was no significant difference between the probiotics and placebo groups in the reduction of patient anxiety scores, even if they are depressive or anxious patients or healthy participants under stress. Subgroup analysis revealed that probiotics had significant effect on depressive symptoms just in patients with depression, and no significant change in anxiety in patients, and no improvement in participant performance under stress.

**Conclusions:** Probiotics could alleviate depressive symptoms in patients with a depression diagnosis or depression scores also in anxiety disorder diagnosis, and suggesting that probiotics may be adjunct therapies for mood or emotional disorders. Therefore, it is essential that probiotics could be more involved in the treatment of patients with depression in the future. The evidence of probiotics successfully treating depression is still insufficient, and more high-quality studies on patients with depression are still needed.

## Introduction

Depression and anxiety disorders are the most common mental disorders in human health. Depression is the main clinical feature of low mood, loss of interest, often accompanied by guilt, hopelessness, loss of appetite and insomnia, and it is one of the main types of mood disorders. Anxiety is an emotional state where the main characteristics are tension, worry, fear, and physical changes such as palpitations, tremors, gastrointestinal tract, respiratory and circulatory disorders without obvious objective causes. The World Health Organization (WHO) estimated that the prevalence of depression in the global population was as high as 4.4% in 2015, and the prevalence of anxiety disorders was estimated to be 3.6% (1). A recent study reported that by combining data from 1 million participants from 30 regions from 1994 to 2014, the one year prevalence of depression was approximately ~7.2% (2). One in nine people worldwide suffered from anxiety in the past year, due to its high prevalence and debilitating features, anxiety disorders ranked sixth among all diseases in the global population (3, 4). Excessive anxiety is associated with many negative health consequences, such as increased risk of coronary heart disease, sleep disorders (5).

In recent years, probiotics have received increasing attention for their extensive clinical applications and beneficial health effects on various clinical disorders including acute and chronic gastrointestinal diseases as well as non-gastrointestinal diseases (6). Previous research has indicated that the intestinal flora plays a more important role in regulating mood and that probiotics have a wider range of therapeutic applications than previously considered (7). Studies have shown that compared with healthy individuals, the composition of intestinal microbiota in patients with major depressive disorders, shows increased levels of phyla *Bacteroidetes, Proteobacteria*, and *Actinobacteria*, and reduced amounts of Firmicutes (8). Due to the small sample size of each study and the heterogeneity of the sample selection, probiotic strains, anxiety assessment scales, and so forth, the effects of probiotics on anxiety showed inconsistent results. Some studies have reported that probiotics were superior to placebo in relieving anxiety (9, 10). However, other studies suggested that there were no differences between the probiotics and placebo (11, 12).

Probiotics can affect mood and host health by regulating the microbial-gut-brain axis (13). Depression, anxiety, and stress are a process of gradually weakening of emotions. Anxiety is a reaction to stress, but persistent and untreated anxiety may lead to deeper mental illnesses like depression (14). Stress usually not only arise from physiologically or emotionally challenging experiences, also from transient reactions in rapid situations. Many studies have found a complicated relationship between human depression and anxiety. Data from Australia data showed that approximately ~57% of people with depression showed comorbid anxiety, and 28% of patients with clinically significant anxiety had depression (15). A Dutch survey showed that 47.5% of patients with major depression also met the criteria for anxiety, and 26.1% of patients with anxiety also met the criteria for major depression (16).

With increasing evidence for the use of probiotics benefitting psychiatric disorders, the number of clinical trials that are examining their application in mental health disorders such as depression, anxiety, and stress has proliferated. Because some meta-analyses have previously reported that probiotics have no significant effect on healthy adults, few studies have included patients with depression (17–19) or anxiety (17) or healthy people but under stress conditions (20). Therefore, this study focused on the effects of probiotics on human symptomatology of depression and anxiety in patients or healthy subject under stress. Motivated by the surge in the number of clinical trials examining this topic, we analyzed the efficacy of beneficial bacteria on depression and anxiety in patients with depression or anxiety disorder diagnosis, and healthy participants under stress.

## Methods

### Search Strategy

Two reviewers searched databases and other sources including PubMed, EMBASE, Medline, and the Cochrane library. Combinations of the following search terms were used: (“depression” OR “mood disorder” OR “anxiety” OR “pressure” OR “under stress”) AND (“probiotics” OR “lactobacillus” OR “bifidobacterium” OR “microbiota” OR “gut bacteria” OR “saccharomyces”). Electronic search supplemented by a manual search retrieved the search terms. Included articles are those published in English-language publications between January 1, 2015 and December 30, 2019. Cohen's kappa was used to assess auditors' consistency in the quality of randomized controlled trials. Statistical analysis was performed using RevMan 5.3 (The Nordic Cochrane Centre, The Cochrane Collaboration, Copenhagen, Denmark) software and SPSS 20.0 (IBM, Armonk, NY, USA). If there is a disagreement between the first two reviewers, a third reviewers participates in decision making.

### Studies Sections and Data Extraction

Eligible studies met the following inclusion criteria:

1. RCT of probiotics for participants with depression, anxiety or under stress;
2. Studies that compared the difference between probiotics and placebos. No limitations on dosage, strain, or form of probiotics;
3. Reports that used similar methods and a scientific rating scale for depression and anxiety e.g., Hospital Anxiety and Depression Scale (HADS) or Beck Depression Inventory (BDI).
4. All participants are adults (age ≥16 years).
5. All participants had no medication history within the 3 months or during the study;

Studies that met any one of the following criteria were excluded:

1. No control group (placebo treatment) in the article;
2. Did not report post-intervention scores on depression and anxiety;
3. Participants who had other diseases.
4. Research on pregnant women.

Data related to the effects of probiotics on depression and anxiety were extracted from all included studies using a table designed by the two reviewers. This table includes the demographic information of included subjects, trial designs, probiotic regimens, rating scales for depression, and anxiety and dropout rate.

### Data Analysis

The analysis was performed with RevMan 5.3. The primary outcome of the study was the standardized mean difference (SMD) of change from baseline in scores on the depression and anxiety rating scales between the probiotics and placebo groups. Statistical heterogeneity was determined by χ^2^ and *I*^2^ statistics. When *P* < 0.05 for the χ^2^ statistic or *I*^2^ > 50%, the heterogeneity was considered high. Therefore, a random-effects model was chosen for meta-analysis. Conversely, in the absence of heterogeneity, a fixed-effect model was used. Due to the relatively high heterogeneity between the studies, a random-effects model was chosen for meta-analysis.

## Results

### Study Selection and Characteristics

**Figure 1:**
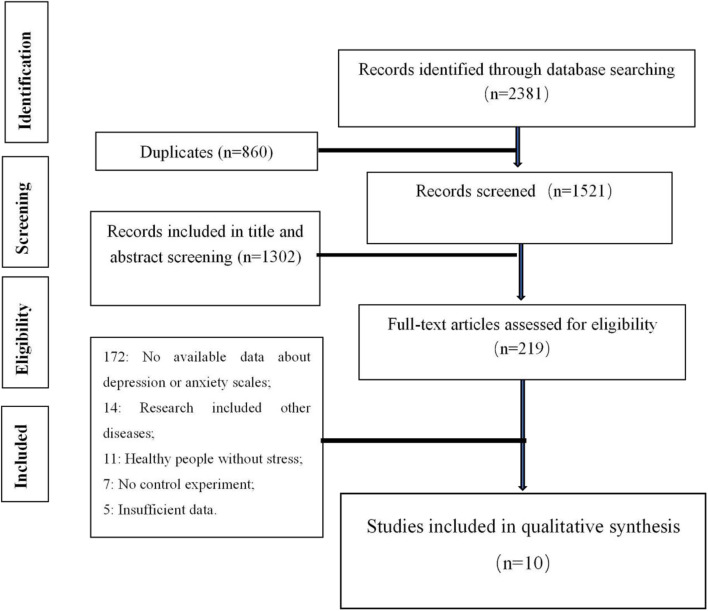
PRISMA flowchart showing the study and selection process of the literature.

A total of four databases were reviewed. After discarding the duplicates, 1,521 studies were remained. The title and abstract screening excluded 1,320 papers with 219 papers remained. The full-text screening further excluded 209 studies, with the remaining 10 studies included in the meta-analysis. Ten clinical trials (involving 344 participants in the probiotics group and 341 participants in the control group) were included based on the inclusion and exclusion criteria. The results of these search processes are described below (Figure 1). Consensus among reviewers regarding the risk of research bias assessment was considered strong consistency (kappa = 0.8).

The characteristics of the 10 studies are shown in Table 1. The publication years ranged from 2016 to 2018. Among the studies, four studies (21–23, 28) were about depression diagnosed participants, one study (29) was about anxiety diagnosed patients, four studies (13, 24, 25, 27) were about academic stress, and the last study (26) was about the mental state of healthy adults after receiving a socially evaluated cold pressor test (SECPT).

**Table 1:** Main characteristics of the included studies.

**References**	**Nation**	**Study sample**	**treatment with placebo/probiotics (men/women)**	**Probiotic dosage and duration**	**Diagnosis criteria**	**Dropout (placebo/conclusions probiotics)**	**Conclusions**
Romijn et al. (21)	Canada	79 adults (age ≥16 years) with at least moderately low mood	39/40; (17/62)	Contained freeze-dried *L. helveticus* R0052 and *B. longum* R0175 bacteria at a dosage of three billion colony-forming units (≥3 × 10^9^ CFU) per 1.5 g sachet for 8 weeks	MADRS; DASS	3/7	No evidence that the probiotic formulation was effective in treating low mood
Rudzki et al. (22)	Poland	79 patients with MDD, mean age of 38.90 years	30/30; (17/43)	For the first 4 weeks of the intervention, patients received 1 capsule (contained 10 × 10^9^CFU of probiotic bacteria *Lactobacillus plantarum* 299v) in the morning and evening for 8 weeks	HAM	9/10	Augmentation of SSRI treatment with probiotic bacteria Lactobacillus Plantarum 299v improved cognitive performance and decreased KYN concentration in MDD patients
Akkasheh et al. (23)	Iran	40 patients aged 20–55 years with MDD	20/20; (6/34)	Patients in the probiotic group received one probiotic capsule containing *Lactobacillus acidophilus* (2 × 10^9^ CFU/g), *Lactobacillus casei* (2 × 10^9^ CFU/g), and *Bifidobacterium bifidum* (2 × 10^9^ CFU/g) daily for 8 weeks	BDI	2/3	Consumption of probiotic supplements improved the BDI scores compared to consumption of the placebo
Takada et al. (24)	Japan	Healthy medical students (average 22.9 years old) with physical symptoms who were undergoing academic stress (*n* = 172)	70/70; (76/64)	Patients received either 100 mL LcS-fermented milk or placebo milk once a day for 8 weeks leading up to the day of the examination	STAI	5/6	There was no significant difference in the changes between both groups at the analyzed points
Takada et al. (25)	Japan	Healthy medical students (average 22.7 years old) with physical symptoms who were undergoing academic stress (*n* = 124)	46/48; (55/39)	Patients received a daily dose of 100 mL LcS-fermented milk or non-fermented placebo milk for 6 weeks	STAI	16/14	No effect of probiotics on the STAI scores
Kelly et al. (26)	Ireland	Healthy adults (*n* = 29) who received SECPTs and were aged between 20 and 33 years of age	15/14; (29/0)	The number of *L. rhamnosus* (JB-1) in the active capsules was 1 × 10^9^ colony-forming units (CFU). Participants were instructed to take one capsule every morning for 8 weeks	BDI; BAI	0/0	No significant effects of probiotics on the BDI scores; *L. rhamnosus* can reduce stress-related behavior and corticosterone release
Nishida et al. (27)	Japan	74 Japanese medical students (average 25.1 years old) preparing for the national examination	37/37; (41/19)	Practitioners ingested CP2305-containing (1 × 10^10^ bacterial cells per 2 tablets) or placebo tablets once daily for 24 weeks	STAI; HADS	6/8	The long-term use of CP2305-containing tablets may improve the mental state, sleep quality, and gut microbiota of healthy adults under stressful conditions
Kato-Kataoka et al. (13)	Japan	Healthy medical students (*n* = 54, average 22.9 years old) undertaking an authorized nationwide. Examination for academic advancement	24/23; (25/22)	A 100-ml bottle of either fermented milk containing *L. casei* strain Shirota or placebo milk was taken daily for 8 weeks	STAI	3/4	The daily consumption of lactic acid bacteria provided a good change of gut microbiota in healthy medical students
Kazemi et al. (28)	Canada	74 patients with MDD (18 < aged <50)	36/38; (23/31)	Contains freeze-dried *L. helveticus* R0052 and *B. longum* R0175 bacteria at a dosage of ten billion colony-forming units (≥10 × 10^9^ CFU) per 5 g sachet, take one sachet each day for 2 months	BDI	10/10	Probiotic supplements to subjects with MDD resulted in an improvement in BDI score
Eskandarzadeh et al. (29)	Iran	Forty-eight drug-free patients (18–65 years old) with a diagnosis of GAD based on DSM-V criteria	24/24; (9/39)	Assigned to two groups to receive daily either one capsule of probiotics (18*10^9^ CFU *Bifidobacterium longum, Bifidobacterium bifidum, Bifidobacterium lactis*, and *Lactobacillus acidophilus* bacteria) or placebo in addition to 25 mg sertraline for 8 weeks	STAI; BAI; HAM	4/8	Probiotics + sertraline combination was superior to sertraline alone in decreasing anxiety symptoms after 8 weeks in patients with GAD

*BAI, Beck Anxiety Inventory; BDI, Beck Depression Inventory; STAI, State-Trait Anxiety Inventory; DASS, Depression Anxiety and Stress Scale; MADRS, Montgomery–Asberg Depression Rating Scale; MDD, major depressive disorder; HAM, Hamilton Depression Rating Scale; HADS, Hospital Anxiety and Depression Scale; PSS, Cohen's Perceived Stress Scale; GAD, generalized anxiety disorder; SECPT, socially evaluated cold pressor test*.

### Methodological Quality and Risk of Bias

The risk of deviation assessment is shown in Table 2. The methodological quality and risk of bias of each study were assessed by the modified Jadad evaluation scale (30). Studies that scored 1–3 were considered low-quality clinical trials, and studies that scored 4–7 were considered high-quality. The data extraction table shows that there are seven high-quality studies (13, 21, 22, 24, 25, 27, 28) and three low-quality studies (23, 26, 29).

**Table 2:** Quality assessment of clinical trials meta-analysis using modified Jadad evaluation scale.

**References**	**Stochastic method (2)**	**Allocation concealment (2)**	**Blind method (1)**	**Exit (1)**	**Baseline similarity (1)**	**Jadad score**
Romijn et al. (21)	Low risk	Low risk	Unclear risk	Low risk	High risk	5
Rudzki et al. (22)	Low risk	Unclear risk	Unclear risk	High risk	Low risk	4
Akkasheh et al. (23)	Unclear risk	High risk	Unclear risk	Low risk	Low risk	3
Takada et al. (24)	Unclear risk	Unclear risk	Unclear risk	Low risk	Low risk	4
Takada et al. (25)	Unclear risk	Unclear risk	Unclear risk	Low risk	Low risk	4
Kelly et al. (26)	Unclear risk	High risk	Unclear risk	Low risk	Low risk	3
Nishida et al. (27)	Unclear risk	Unclear risk	Unclear risk	Low risk	Low risk	4
Kato-Kataoka et al. (13)	Unclear risk	Unclear risk	Unclear risk	Low risk	Low risk	4
Kazemi et al. (28)	Low risk	Low risk	Unclear risk	Low risk	Low risk	6
Eskandarzadeh et al. (29)	Unclear risk	Unclear risk	Unclear risk	High risk	Low risk	3

### The Effects of Probiotics on Patients With Depression or Anxiety, and Participants Under Stress

**Figure 2:**
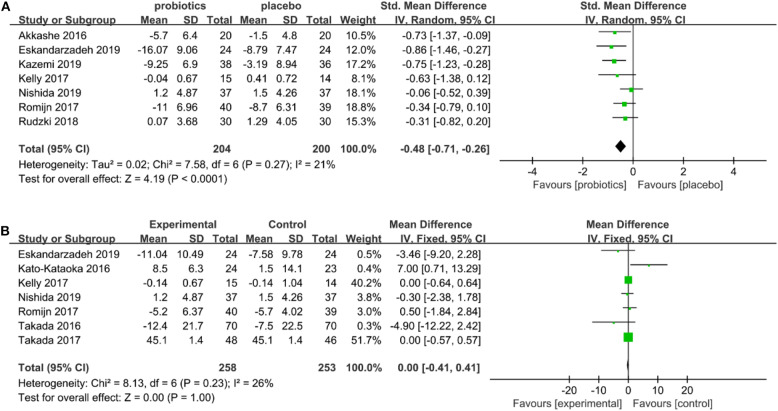
**(A)** Forest plots for depression status. **(B)** Forest plots for anxiety status.

Seven studies include depression scales with 204 subjects in the probiotics group and 200 subjects in the placebo group. Due to the heterogeneity of the included studies (*I*^2^ = 21%; *P* = 0.003 for χ^2^-test), the random-effects model was applied. The meta-analysis showed significant differences in the relief of depression scores between the probiotics and placebo groups (SMD = −0.48, 95% CI: −0.71 to −0.26, *P* = 0.27; Figure 2A). The results showed that probiotics have obvious effects on positive changes in depressive symptomatology related scores. The forest plots of the meta-analysis are shown in Figure 2A.

Seven studies include anxiety scales with 258 subjects in the probiotics group and 253 subjects in the placebo group. The included studies had heterogeneity (*I*^2^ = 26%; *P* =0.23 for χ^2^-test), so the random-effects model was applied. Meta-analysis shows that there was no significant difference between the probiotics group and placebo group (SMD = 0.00, 95% CI: −0.41 to 0.41, *P* = 0.23; Figure 2B) in alleviating anxiety scores. The meta-analysis of forest maps is shown in Figure 2B.

### Subgroup Analyses

**Figure 3:**
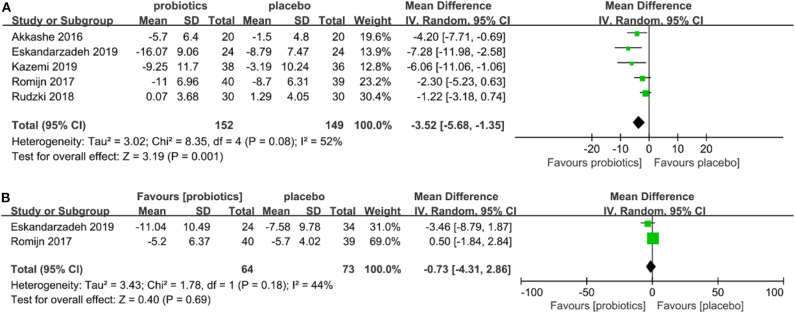
**(A)** Forest plot showing depressive symptoms of probiotics in patients with depression or anxiety. **(B)** Forest plot showing anxiety symptoms of probiotics in patients with depression or anxiety.

**Figure 4:**
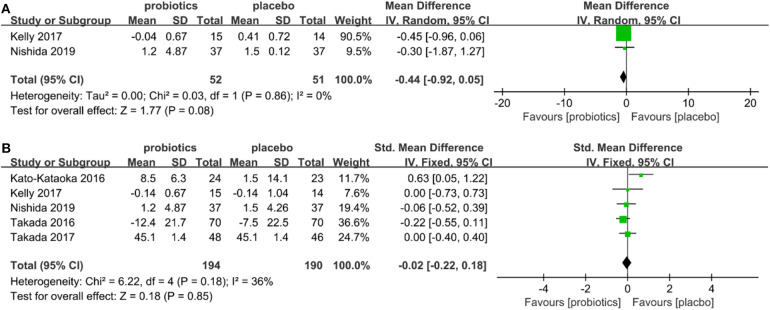
**(A)** Forest plot showing subgroup analysis of depression in individuals with under stress. **(B)** Forest plot showing subgroup analysis of anxiety in individuals with under stress.

Two subgroup analyses were performed: (a) individual subgroups of patients with a diagnosis of depression and anxiety disorder (Figures 3A,B); (b) healthy individuals under stress (Figures 4A,B) were analyzed. The results showed that probiotics had significantly reduced depressive symptom scores improved depressive symptoms (SMD = −3.52, 95% CI −5.68 to −1.35, *P* = 0.08; Figure 3A), but had no significant effect on anxiety scores (SMD = −0.73, 95% CI −4.31 to 2.86, *P* = 0.18; Figure 3B) in patients with depression or anxiety diagnosis. There was no significant difference in depression scores (SMD = −0.44, 95% CI −0.92 to 0.05, *P* = 0.56; Figure 4A) for individuals under stress, and no significantly effect on anxiety scores (SMD = 0.02, 95% CI −0.22 to 0.18, *P* = 0.18; Figure 4B).

### Publication Bias

**Figure 5:**
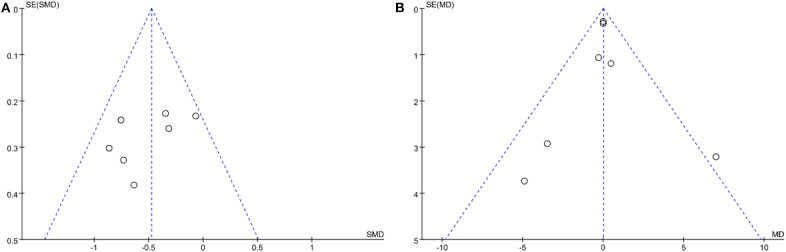
**(A)** Funnel plot of depression. **(B)** Funnel plot of anxiety.

Funnel plot was used to assess publication bias qualitatively. The funnel plot (Figure 5A) shows that depression studies were basically symmetrically distributed without any evidence of publication bias. The funnel plot (Figure 5B) shows that the anxiety researches were partially symmetrical, so the possibility of publication bias was very small.

## Discussion

### Summary of Main Findings

In this study, the meta-analysis showed that there were significantly better depression scores in the probiotic group than in the placebo group (SMD = −0.47, 95% CI: −0.67 to −0.27, *P* = 0.27). However, there was no significant difference between the probiotics group and the placebo group (SMD = 0.00, 95% CI: −0.41 to 0.41, *P* = 0.23) in alleviating anxiety scores. Subgroup analysis further found that probiotics could improve depression scores in patients with depression or anxiety diagnoses (SMD = −3.52, 95% CI −5.68 to −1.35, *P* = 0.08), but did not affect patients' anxiety scores (SMD = −0.73, 95% CI −4.31 to 2.86, *P* = 0.18). Furthermore, we have found that probiotics do not improve nor depression scores (SMD = −0.44, 95% CI −0.92 to 0.05, *P* = 0.56), nor anxiety scores (SMD = 0.02, 95% CI −0.22 to 0.18, *P* = 0.18) in participants under stress. These results were not exactly the same as those of the previous meta-analysis. A paper (31) with five randomized clinical trials found that the effects of probiotics on mood were statistically significant in both depressed and healthy individuals. One study (19) showed that the overall effect of probiotics in depressed and healthy individuals was statistically irrelevant, but a statistically significant benefit was observed in patients with mild to moderate depression. Recent studies (32) found that probiotics were effective for depression and anxiety. The difference in results is mainly due to the different criteria for inclusion in the study, there were very few patients with depression in these meta-analyses, and they were mostly conducted with healthy people or other disease groups; there is no research on the treatment effects of probiotics for depression and anxiety in depressive patients. Of course, there were reports consistent with the results of this review. One study (33) found that probiotics could have a significant therapeutic effect on subjects with depression. Another study conducted by Liu et al. (17) also found no significant differences between probiotics and placebo in relieving anxiety. In a subgroup analysis of patients with depression or anxiety, five studies (21–23, 26, 28) were found to show significant effects of probiotics on depression scores. Studies of anxiety symptoms in depression or anxiety patients found that four studies (13, 21, 25, 26) showed no significant differences, and only three studies (25, 27, 29) showed that probiotics can reduce anxiety scores in patients. The study of anxiety symptoms of participants under stress found that only two studies (25, 27) showed that probiotics could relieve anxiety scores in participant performance under stress. Therefore, it is essential that probiotics could be more involved in the treatment of patients with depression in the future. In addition, according to the main characteristics of the included studies, it is found that the treatment of depression often requires long-term treatment.

### Strengths and Limitations

This study differs from other studies in that it has clear and strict research selection criteria. Previous reviews covering all human studies, regardless of their disease state, with limited value in determining the clinical efficacy of probiotics in the treatment of healthy people under stress or clinically diagnosed people under depressive or anxiety disorders. This study focused on the effects of probiotics on participants with different emotional states in depression, anxiety, and stress.

In this study, 10 randomized controlled trials were published in the past 5 years. From the forest plot of depression and anxiety, probiotics are significantly more effective in treating depression than anxiety. Subgroup analyses showed that probiotics were more effective in treating patients with depression and anxiety than individuals under stress. One study on depression (23) with larger sample sizes showed statistical significance between the probiotics group and the placebo group. Two studies (21, 29) showed that probiotics had no statistically significant effect on anxiety scores of participants with depression or anxiety. Two studies (26, 27) show that probiotics have no effect on individuals' depression scores and anxiety scores under stress. However, there were some limitations to this research, including differences between the included studies in the criteria for depression, the heterogeneity of the intervention, and the bias involved in the trial. There are some other limitations to this study. First, the study of probiotics for the treatment of depression is in its infancy, and the number of corresponding studies is small. Second, within each included study, the selected probiotics, dosage and treatment methods are heterogeneous and may interfere with the results of the overall study. Finally, differences in depression and anxiety scales still cause large heterogeneity.

### Conclusions

In conclusion, the meta-analysis showed a significantly lower depression score in the probiotic group compared to the placebo group (SMD = −0.47, 95% CI: −0.67 to −0.27, *P* = 0.27). There was no significant difference between the probiotic group and the placebo group in alleviating anxiety scores. The subgroup analyses found that probiotics had a significant effect on the scores of patients with depression and anxiety, suggesting that probiotics may be adjunct therapies for deep mental illness. Larger studies are needed in well-defined clinical populations to determine the clinical utility of this novel treatment, and to further investigate potential underlying mechanisms.

## Data Availability Statement

All datasets generated for this study are included in the article/supplementary material.

## Author Contributions

LC, CL, and SS contributed to the search strategy, data extraction, and preparation of the first draft of the manuscript. LC, YL, WL, WC, LY, JZ, AG, ZL, and SG contributed to writing, reviewing, or revising the paper.

## Conflict of Interest

The authors declare that the research was conducted in the absence of any commercial or financial relationships that could be construed as a potential conflict of interest.
